# Multisystem Inflammatory Syndrome in Children (MIS-C) With Hematological Manifestations: A Case Report

**DOI:** 10.7759/cureus.24035

**Published:** 2022-04-11

**Authors:** Yazeid Alrefaey, Ahmad Alamoudi, Reshale Johar, Rawia F Albar

**Affiliations:** 1 College of Medicine, King Saud Bin Abdulaziz University for Health Sciences, Makkah, SAU; 2 College of Medicine, King Saud Bin Abdulaziz University for Health Sciences, Jeddah, SAU; 3 Pediatrics, King Abdulaziz Medical City, Jeddah, SAU

**Keywords:** pediatric inflammatory multisystem syndrome, coronavirus disease 2019 (covid-19), kawasaki disease mimic, mis-c, covid 19

## Abstract

Multisystem inflammatory syndrome in children (MIS-C) is a new emerging severe form of coronavirus disease 2019 (COVID-19) that recently has been recognized in April 2020 in the United States and the United Kingdom. MIS-C is an uncommon condition that mainly affects children who previously had a COVID-19 infection, and it can have serious outcomes if left untreated properly. The full clinical spectrum of this disease is yet not fully determined or understood. Here, we report a case of a 12-year-old girl, previously medically free, who presented to the emergency room complaining of shortness of breath and dizziness for two days. The patient was confirmed to have a COVID-19 infection in the workup. Laboratory studies showed elevated inflammatory markers, leukopenia, and elevated liver enzymes. Upon admission, the patient developed a persistent fever with a spike of 40ºC, not resolved with antibiotics or anti-inflammatory drugs. This was managed with intravenous immunoglobulin (IVIG) followed by steroids but did not show dramatic change initially. The patient eventually improved with management and was discharged.

## Introduction

Following the emergence of the coronavirus disease 2019 (COVID-19) pandemic caused by severe acute respiratory syndrome coronavirus 2 (SARS-CoV-2), the clinical implications of the virus on the pediatric population were mild and non-fatal in most cases. It was thereby thought that children were highly resilient and at low risk. However, unusual presentations of children who had recently been infected with COVID-19 were noted. These patients displayed signs and symptoms of a multisystem inflammatory syndrome (MIS) after the acute phase of COVID-19 infection had resolved [1-2]. Multisystemic inflammatory syndrome in children is an inflammatory condition that is capable of affecting virtually any organ system. It was first recognized in April of 2020 when the National Health Service in the United Kingdom reported cases of fever, shock, severe gastrointestinal pain, and cardiac dysfunction. MIS-C may begin weeks after a child is infected with SARS-CoV-2 [2-3]. Despite its poorly understood pathogenesis, MIS-C greatly resembles Kawasaki disease (KD), and both MIS-C and KD are part of the SARS-CoV-2-related inflammatory illness spectrum. Some of the shared features between them include fever, mucocutaneous involvement, including but not limited to rashes and conjunctival injection, cardiac involvement, and elevated inflammatory markers. Additionally, KD's standard intravenous immunoglobulin (IVIG) treatment has also been utilized and found to be effective in MIS-C [4-5]. Hence, all of the aforementioned points along with the notable variation in phenotypes and severity highlight the difficulty of diagnosing MIS-C [6]. As for the labs, a study published in 2021 concluded that lymphopenia was an independent predictor of MIS-C and differentiates between both diagnoses as well as other inflammatory markers [5].

## Case presentation

This is a case of a 12-year-old female patient who presented to the emergency department with shortness of breath, paroxysmal nocturnal dyspnea, and orthopnea, associated with dizziness, decreased activity, and oral intake with elbow pain for two days. The patient was previously healthy, with a history of adenotonsillectomy three years ago. There was a previous history of upper respiratory tract infection with a fever two weeks ago. Upon thorough infection workup, the patient is confirmed to have COVID-19 immunoglobulin G (IgG)-positive, with the exclusion of other infections. She was admitted to the general pediatric ward as a case of multisystem inflammatory syndrome in children (MIS-C).

The patient came vitally stable with the exception that she was tachycardic with 140 beats per minute. Blood gas, electrocardiograph, and chest X-ray were all normal. Labs showed leukopenia, lymphopenia, neutropenia, mild D-dimer and fibrinogen elevation, elevated liver enzymes, and elevated inflammatory markers, as shown in Table 1. A CT pulmonary angiogram and echocardiogram showed normal results. Multiple lymph nodes were swelling in the left axillary and subpectoral areas. Bone marrow aspiration was done for further investigation.

**Table 1:** The abnormal results of this patient on the day of presentation

Investigation	Patient lab	Reference range
Complete blood count		
Hemoglobin	9.1	11-14.5 g/dl
White blood cell (WBC)	2	4-12x10^9/L
Lymphocyte	0.52	1.5-4x10^9/L
Neutrophil	1.59	2-7.5x10^9/L
D-dimer	1.8	<0.4 U/mL
Fibrinogen	4.2	2-4 g/L
Inflammatory markers		
Procalcitonin (PRC)	0.34	0.25 ug/L
Lactate dehydrogenase (LDH)	1035	157-272 U/L
Ferritin	1389	13.7-78.8 ug/L
C-reactive protein (CRP)	5.2	0-5 mg/L
Erythrocyte sedimentation rate (ESR)	87	0-20 mm/hr
Liver enzymes		
Gamma-glutamyltransferase	90	7-21 IU/L
Alanine transaminase	61	9-25 U/L
Aspartate aminotransferase	105	13-26 IU/L

The day after admission, the patient was with persistent fever, a spike of 39.2ºC, along with non-purulent conjunctivitis. At this moment, we started treatment with antibiotics and a single infusion over eight hours of intravenous immunoglobin (IVIG) 2 g/kg. Although IVIG was used, there was no dramatic response in terms of complete blood count, inflammatory markers, and fever, so the second dose was administered after four days of the first dose along with acetaminophen, but the fever was persistent with a spike of 40.2ºC, along with multiple vomiting episodes during the course. Three days after the second IVIG, her condition improved and she was afebrile. After 24 hours with no fever, the inflammatory markers were high so two pulses of steroid 2 mg/kg/day were given one day apart. Complete blood count (CBC) showed improvement and returned to the normal range except for the neutropenia 1.51x10^9/L. Also, inflammatory markers and liver enzymes improved and returned to the normal ranges. The bone marrow result shows hypocellular marrow with no overt morphological evidence of malignancy. The patient was afebrile and hemodynamically stable after that and was then discharged. A few days later after discharge, the patient developed a maculopapular rash over the cheeks and arms that was not related to any medication; this then resolved after a couple of days with no management.

## Discussion

MIS-C is a severe form of COVID-19 that is characterized by severe progressive symptoms with yet unknown pathogenesis. Researchers concluded that MIS-C is a different variant of the severe form of COVID-19 [7]. Children with MIS-C usually do not present with respiratory manifestations as cases of COVID-19 [8]. Classical manifestation typically occurs after a few weeks of COVID-19 infection with positive antibodies. According to the Centers for Disease Control and Prevention (CDC), pediatric age group, persisting fever, laboratory evidence of high inflammatory markers, signs or symptoms of multisystem involvement, severe illness that requires hospitalization, and clinically confirmed COVID-19 infection or exposure are a must to diagnose patients with MIS-C [9]. This patient fulfilled the definition and criteria of MIS-C.

The majority of patients of MIS-C were previously healthy school-aged children. A systematic review of published case studies of a total of 270 reports found that all children with MIS-C report fever and gastrointestinal symptoms in most of the cases. Symptoms of Kawasaki disease such as shock, rash, conjunctivitis, oral mucosal changes, and limb changes are common in cases of MIS-C and were reported in almost 50% of the cases. Respiratory symptoms like cough were uncommon findings and shock was reported in a third of the patients; the majority were cardiogenic. Lymphadenopathy was present in many cases [8]. Our case presented with shortness of breath, dizziness, decreased activity and feeding, elbow pain, and lymphadenopathy. During admission and after discharge, some similar manifestations appeared like persistent fever, vomiting, non-purulent conjunctivitis, and skin rash. All of the symptoms mentioned improved after proper management.

In the same systematic review study, laboratory examination reported the following: low hemoglobin, elevated leukocytes with mainly neutrophils, which point to inflammation, along with a decrease in lymphocytes [8]. In our case, the patient showed low hemoglobin, leukopenia, lymphopenia, and neutropenia, which is not consistent with most cases; it is worth mentioning that all of them improved after management except neutrophil count. Inflammatory markers, such as C-reactive protein (CRP), procalcitonin (PCT), erythrocyte sedimentation rate (ESR), and interleukin-6 (IL-6) are also increased [8]. Also, ferritin was high in our case (1389 ug/L); one research conducted showed that patients with a ferritin level of 558 to 1,176 ng/mL are a major indicator for severe cases [10]. Moreover, raised D-dimer level, lactic acid dehydrogenase, and troponin I have been demonstrated in many cases of MIS-C. Similarly, our patient presented with elevated D-dimer and fibrinogen, which occur in 70-100% of the cases according to the CDC [9]. One meta-analysis concluded that inflammatory markers, especially WBC, CRP, ferritin, D-dimer, lactate dehydrogenase (LDH), fibrinogen, and ESR levels, were correlated with MIS-C [11]. Elevated liver enzymes had been previously demonstrated in a few cases [12]. In this case of MIS-C, hematological systems are involved in the shape of leukopenia, lymphopenia, and neutropenia, however, after proper management, labs improved except for isolated neutropenia.

The patient was covered with antibiotics and treated for possible MIS-C. According to a recent literature review, the majority of patients with multisystem inflammatory syndrome recovered from treatment with intensive care, which included inotropes, respiratory support, intravenous immunoglobulin (IVIG), and steroids [8,13]. One study showed that out of 78% of those who received IVIG infusion, 25% of them reported IVIG resistance and required a second dose of IVIG or IVIG given with glucocorticoids. In comparison to typical KD, resistance to IVIG in MIS-C patients was significantly higher [14]. In our case, the patient resolved after a couple of days of two doses of IVIG administration following steroid after the fever subsided, unlike most cases, which showed prompt and dramatic improvement in fever and laboratory investigations. More management options can improve the quality of life of such cases that showed resistance to this management.

## Conclusions

MIS-C is a severe form of COVID-19; yet, until now, there is no clear pathogenesis and curative treatment. Here, we present a case with some uncommon presentations for further exploring the sign spectrum of this disease. Although IVIG administration showed improvement in many cases, few cases showed resistance to this management plan, like our case. Hence, more study of the treatment protocols can improve the quality of life for resistant cases. Finally, we encourage early recognition of MIS-C as a differential of patients who have recently been infected with COVID-19 for a better critical care approach.
